# Long-term voluntary running prevents the onset of symptomatic *Friedreich’s ataxia* in mice

**DOI:** 10.1038/s41598-020-62952-6

**Published:** 2020-04-08

**Authors:** Henan Zhao, Bevan M. Lewellen, Rebecca J. Wilson, Di Cui, Joshua C. Drake, Mei Zhang, Zhen Yan

**Affiliations:** 1 Department of Medicine, University of Virginia School of Medicine, Charlottesville, Virginia 22908 USA; 2 Department of Pharmacology, University of Virginia School of Medicine, Charlottesville, Virginia 22908 USA; 3 Department of Molecular Physiology and Biological Physics, University of Virginia School of Medicine, Charlottesville, Virginia 22908 USA; 4 Center for Skeletal Muscle Research at Robert M. Berne Cardiovascular Research Center, University of Virginia School of Medicine, Charlottesville, Virginia 22908 USA; 5 Dalian Medical University, Dalian, Liaoning 116044 China

**Keywords:** Mitochondria, Cardiomyopathies, Metabolic disorders

## Abstract

The common clinical symptoms of *Friedreich’s* ataxia (FRDA) include ataxia, muscle weakness, type 2 diabetes and heart failure, which are caused by impaired mitochondrial function due to the loss of frataxin (FXN) expression. Endurance exercise is the most powerful intervention for promoting mitochondrial function; however, its impact on FRDA has not been studied. Here we found that mice with genetic knockout and knock-in of the *Fxn* gene (*KIKO* mice) developed exercise intolerance, glucose intolerance and moderate cardiac dysfunction at 6 months of age. These abnormalities were associated with impaired mitochondrial respiratory function concurrent with reduced iron regulatory protein 1 (Irp1) expression as well as increased oxidative stress, which were not due to loss of mitochondrial content and antioxidant enzyme expression. Importantly, long-term (4 months) voluntary running in *KIKO* mice starting at a young age (2 months) completely prevented the functional abnormalities along with restored Irp1 expression, improved mitochondrial function and reduced oxidative stress in skeletal muscle without restoring Fxn expression. We conclude that endurance exercise training prevents symptomatic onset of FRDA in mice associated with improved mitochondrial function and reduced oxidative stress. These preclinical findings may pave the way for clinical studies of the impact of endurance exercise in FRDA patients.

## Introduction

*Friedreich’s* ataxia (FRDA) is the most common autosomal recessive ataxia in the Caucasian population^1–4^ with detrimental clinical symptoms, including ataxia, muscle weakness, type 2 diabetes and heart failure^5,6^. These symptoms usually first appear in childhood or adolescence and worsen over time. A hypertrophic cardiomyopathy is an important clinical trait, which contributes significantly to disability and early death^7,8^. A high percentage of FRDA patients have glucose intolerance or diabetes mellitus^4^, and exercise capacity is usually severely diminished^9^, leading to wheelchair binding within 10 to 20 years after the disease onset^10^.

FRDA is caused by GAA repeat expansions in both alleles of the frataxin (*FXN*) gene within intron 1 located in chromosome 9^11,12^ from normally around 5–30 to >90^6,12^. The GAA expansion mutations lead to reduced expression of frataxin, a mitochondrial protein, which is involved in assembly of iron-sulfur clusters (ISC) and/or function as an iron chaperone or an iron storage protein^13^. Although the precise function of frataxin is not entirely clear yet, substantial evidence shows that frataxin deficiency leads to inefficient use of iron during ISC synthesis, inhibition of ISC formation and/or increased production of reactive oxygen species (ROS) via the Fenton reaction, resulting in iron deposition, mitochondrial dysfunction and oxidative damage^14,15^. It is these abnormal functions in the metabolically active tissues/organs that eventually lead to the onset of clinical symptoms. Unfortunately, no pharmacologic treatment up to date has been proven to impede FRDA progression effectively.

Exercise training is one of the most powerful means for improving mitochondrial function and antioxidant defense in various tissues/organs in healthy humans and animal models^16–20^. However, whether endurance exercise would be beneficial to FRDA patients is unpredictable as endurance exercise may cause temporary oxidative stress and mitochondrial damage^21,22^. Even if endurance exercise proves to be beneficial, it may not be effective at all phases of the disease as the disease status of the patient may dictate the outcome of the exercise intervention, and thus it would be important to determine the appropriate time to start exercise training. Ironically, one of the most significant clinical symptoms of FRDA is exercise intolerance^9^, which may be due to the primary defects in the cardiopulmonary and skeletal muscle systems, and secondary deconditioning caused by neuronal and other defects. A biologically important and clinically relevant question is whether exercise training would reverse and/or prevent the pathological processes of FRDA. Unfortunately, there has not been systematic research on the impact of exercise on FRDA in animal models or in human patients.

The present research sought to investigate the impacts of endurance exercise in a mouse model of FRDA. We investigated exercise capacity, cardiac function and metabolic function as well as the expression of frataxin and its functional regulators in various tissues at different ages in *Fxn*^*tm1Mkn*^*Fxn*^*tm1Pand*^ mice (frataxin knock-in/knockout mice, KIKO). We also subjected KIKO mice to long-term voluntary running and investigated the impact of endurance exercise and dissected the potential mechanisms that might underlie the impacts of exercise

## Results

### Age-dependent exercise intolerance, cardiac dysfunction and metabolic abnormality in KIKO mice

**Figure 1:**
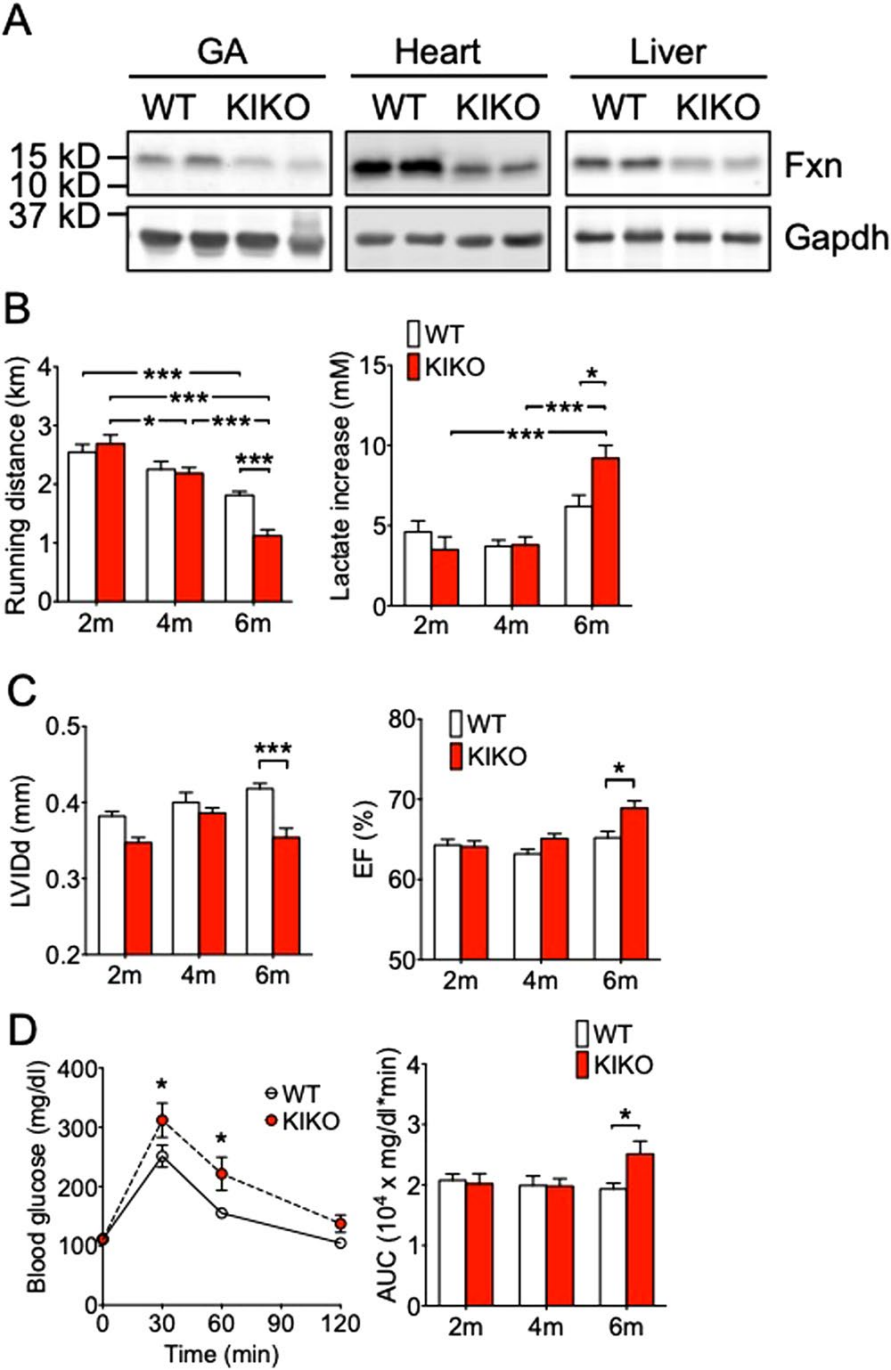
Age-dependent development of exercise intolerance, cardiac dysfunction and glucose intolerance in KIKO mice. Treadmill running test, echocardiography and glucose tolerance test were performed in KIKO and WT littermates at 2 (n = 14–21), 4 (n = 13–25) and 6 months (n = 18–36) of age. (**A)** Representative western blot images for Fxn in gastrocnemius muscle, heart and liver at 6 months of age. A ~50% reduction was detected in all these tissues in KIKO mice compared with WT mice (also shown in Supplemental Fig. S1B); (**B**) Running distance and blood lactate increase in KIKO and WT mice by treadmill running test; (**C**) Echocardiography measurements of left ventricular internal diameter end diastole (LVIDd) and ejection fraction (EF) in KIKO and WT mice; and (**D**) Blood glucose levels during GTT and the area under the curve (AUC) in KIKO and WT mice. *, ** and *** denote *P* < 0.05, *P* < 0.01 and *P* < 0.001, respectively.

We assessed muscle strength, cardiac function, exercise capacity as well as whole body glucose metabolism in KIKO mice on the premise that if KIKO mice recapitulate FRDA, we would be able to detect changes in these functional parameters in an age-dependent manner. We first confirmed the genotype by immunoblotting for all mice that have been genotyped by PCR of tail DNA (Supplemental Fig. S1A). We confirmed that KIKO mice have reduced frataxin protein expression at ~50% of the levels in WT mice in skeletal muscle, heart and liver at 2, 4 (Supplemental S1B) and 6 months of age (Fig. 1A). The tibia length and body weight were similar between WT and KIKO mice (Supplemental Fig. S1C, Table S1), suggesting that there was no growth retardation in KIKO mice. WT mice showed moderate decline in treadmill running distance as they aged with a trend of greater increase of blood lactate at exhaustion at 6 months of age (Fig. 1B). KIKO mice had similar running distance and blood lactate increase at 2 and 4 month of age compared with WT mice, but had significantly reduced running distance and greater increase of blood lactate following treadmill running at 6 months of age (*p* < 0.001 and *p* < 0.05, respectively, vs. WT mice) (Fig. 1B). These findings suggest that KIKO mice have reduced Fxn expression early in life, but do not develop exercise intolerance until 6 months of age. WT and KIKO mice showed no differences in muscle mass (Supplemental Table S1), but both WT and KIKO mice had a gradual, age-dependent decline of twitch and tetanic forces of posterior hindlimb muscles to the same degree (Supplemental Fig. S1D). These findings ruled out the possibility that the age-dependent development of exercise intolerance in KIKO mice is due to preferential loss of muscle mass or strength.

To determine if KIKO mice develop age-dependent cardiac dysfunction as shown in FRDA patients, we performed serial electrocardiography (ECG) and echocardiography in KIKO and WT mice. ECG showed no significant differences in all parameters measured, including heart rate, heart rate variability, PR, QRS and QT intervals, between KIKO and WT mice at 2, 4 and 6 months of age (Supplemental Table S2). There were no significant differences in echocardiography parameters between KIKO and WT mice at 2 and 4 months of age (Tables 1 and 2). However, majority of the parameters (increased IVSd, IVSs, FS and EF and decreased LVIDd, LVIDs and PWT) changed significantly at the 6 months age. In particular, reduced LVIDd indicates diastolic dysfunction, while there was compensated cardiac function as shown by moderately increased EF and FS (Fig. 1C and Table 1). Therefore, KIKO mice developed diastolic dysfunction with preserved function by 6 months of age.

**Table 1:** KIKO mice develop cardiac dysfunction at 6 months of age measured by echocardiogram.

	2 months		4 months		6 months	
	WT	KIKO	WT	KIKO	WT	KIKO
	(n = 21)	(n = 14)	(n = 13)	(n = 25)	(n = 35)	(n = 14)
IVSd (mm)	0.076 ± 0.003	0.081 ± 0.003	0.089 ± 0.003	0.085 ± 0.002	0.083 ± 0.002	0.099 ± 0.004***
LVIDd (mm)	0.382 ± 0.006	0.347 ± 0.007	0.400 ± 0.013	0.386 ± 0.007	0.418 ± 0.007	0.354 ± 0.012***
PWd (mm)	0.086 ± 0.004	0.082 ± 0.005	0.072 ± 0.004	0.077 ± 0.002	0.071 ± 0.002	0.088 ± 0.005**
IVSs (mm)	0.115 ± 0.004	0.115 ± 0.003	0.124 ± 0.005	0.125 ± 0.003	0.121 ± 0.003	0.143 ± 0.005**
LVIDs (mm)	0.271 ± 0.005	0.246 ± 0.005	0.287 ± 0.010	0.272 ± 0.006	0.294 ± 0.006	0.240 ± 0.009***
PWs (mm)	0.123 ± 0.005	0.111 ± 0.005	0.108 ± 0.006	0.110 ± 0.003	0.109 ± 0.003	0.119 ± 0.006
PWT (%)	45.8 ± 4.6	36.6 ± 3.7	53.2 ± 7.4	43.5 ± 3.4	56.2 ± 3.3	37.2 ± 4.4*
FS (%)	29.1 ± 0.5	29.0 ± 0.5	28.4 ± 0.4	29.7 ± 0.4	29.8 ± 0.6	32.3 ± 0.6*
EF (%)	64.3 ± 0.7	64.1 ± 0.7	63.2 ± 0.6	65.1 ± 0.6	65.2 ± 0.8	68.9 ± 0.9*

IVS, interventricular septal thickness; LVID, left ventricular internal dimension; PW, posterior wall thickness; d, end diastole; s, end systole; PWT, posterior wall thickening; EF, ejection fraction; FS, fractional shortening. Values are presented as mean ± SE. *, ** and *** denote p < 0.05, p < 0.01 and p < 0.001 (vs. WT of the same age), respectively.

**Table 2:** Impact of 4-week of voluntary running on cardiac function in WT and KIKO mice at 2 or 4 months of age.

	WT		KIKO	
	Sed	Ex	Sed	Ex
**2 months of age**				
	**(n = 13)**	**(n = 8)**	**(n = 8)**	**(n = 8)**
IVSd (mm)	0.078 ± 0.003	0.081 ± 0.004	0.091 ± 0.004	0.083 ± 0.003
LVIDd (mm)	0.420 ± 0.008	0.398 ± 0.008	0.371 ± 0.008^+++^	0.384 ± 0.008
PWd (mm)	0.074 ± 0.003	0.073 ± 0.003	0.067 ± 0.005	0.084 ± 0.004*
IVSs (mm)	0.118 ± 0.005	0.121 ± 0.006	0.118 ± 0.005	0.120 ± 0.003
LVIDs (mm)	0.300 ± 0.008	0.264 ± 0.007**	0.266 ± 0.007^++^	0.260 ± 0.008
PWs (mm)	0.112 ± 0.004	0.118 ± 0.007	0.098 ± 0.008	0.123 ± 0.006
PWT (%)	52.5 ± 4.4	62.6 ± 6.3	46.3 ± 9.6	47.1 ± 6.4
FS (%)	28.6 ± 0.6	33.9 ± 0.8***	28.4 ± 0.4	32.4 ± 1.2*
EF (%)	63.6 ± 0.9	71.0 ± 1.1***	63.2 ± 0.6	68.9 ± 1.8*
**4 months of age**				
	**(n = 6)**	**(n = 6)**	**(n = 6)**	**(n = 7)**
IVSd (mm)	0.092 ± 0.007	0.089 ± 0.005	0.091 ± 0.004	0.095 ± 0.005
LVIDd (mm)	0.422 ± 0.012	0.422 ± 0.011	0.388 ± 0.016	0.380 ± 0.008
PWd (mm)	0.064 ± 0.002	0.072 ± 0.003	0.076 ± 0.010	0.087 ± 0.007
IVSs (mm)	0.126 ± 0.007	0.135 ± 0.006	0.123 ± 0.005	0.129 ± 0.006
LVIDs (mm)	0.302 ± 0.008	0.296 ± 0.008	0.273 ± 0.014	0.256 ± 0.007^+^
PWs (mm)	0.099 ± 0.005	0.103 ± 0.003	0.121 ± 0.011	0.130 ± 0.005^+^
PWT (%)	55.6 ± 4.5	44.6 ± 3.1	63.6 ± 7.1	52.0 ± 7.8
FS (%)	28.6 ± 0.5	30.0 ± 0.4	29.7 ± 0.8	32.8 ± 0.6^+^**
EF (%)	63.6 ± 0.7	65.7 ± 0.6	65.2 ± 1.1	69.5 ± 0.8^+^**

IVS, interventricular septal thickness; LVID, left ventricular internal dimension; PW, posterior wall thickness; d, end diastole; s, end systole; PWT, posterior wall thickening; EF, ejection fraction; FS, fractional shortening. Values are presented as mean ± SE. *, ** and *** denote p < 0.05, p < 0.01 and p < 0.001 (between Sed and Ex groups of the same genotype), respectively. +, ++ and + ++ denote p < 0.05, p < 0.01 and p < 0.001 (between two genotypes), respectively.

To assess metabolic function, we performed glucose tolerance test (GTT) in KIKO and WT mice at different ages. There were no significant differences in GTT parameters between KIKO and WT mice at 2 and 4 months (Supplemental Fig. S1E), but 6-month-old KIKO mice showed significant glucose intolerance (*P* < 0.01 at 30 and 60 min time points in GTT; Fig. 1D) with a greater area under the curve (AUC) than WT mice (*P* < 0.01; Fig. 1D). These findings support that KIKO mice develop age-dependent insulin resistance with impaired whole-body glucose clearance.

To determine if the functional abnormalities in KIKO mice are due to reduced expression of mitochondrial proteins, we assessed the expression of cytochrome oxidase 4 (Cox4) and electron transport chain complexes (complex I (CI), complex II (CII), complex III (CIII), and complex V (CV)) expressions in skeletal muscle, heart and liver. We found no evidence of significantly altered levels of these proteins between KIKO and WT mice at 6 months of age (Supplemental Fig. S1F). Therefore, the functional abnormalities are not due to reduced mitochondrial respiratory protein expression.

### KIKO mice adapt to voluntary running normally

**Figure 2:**
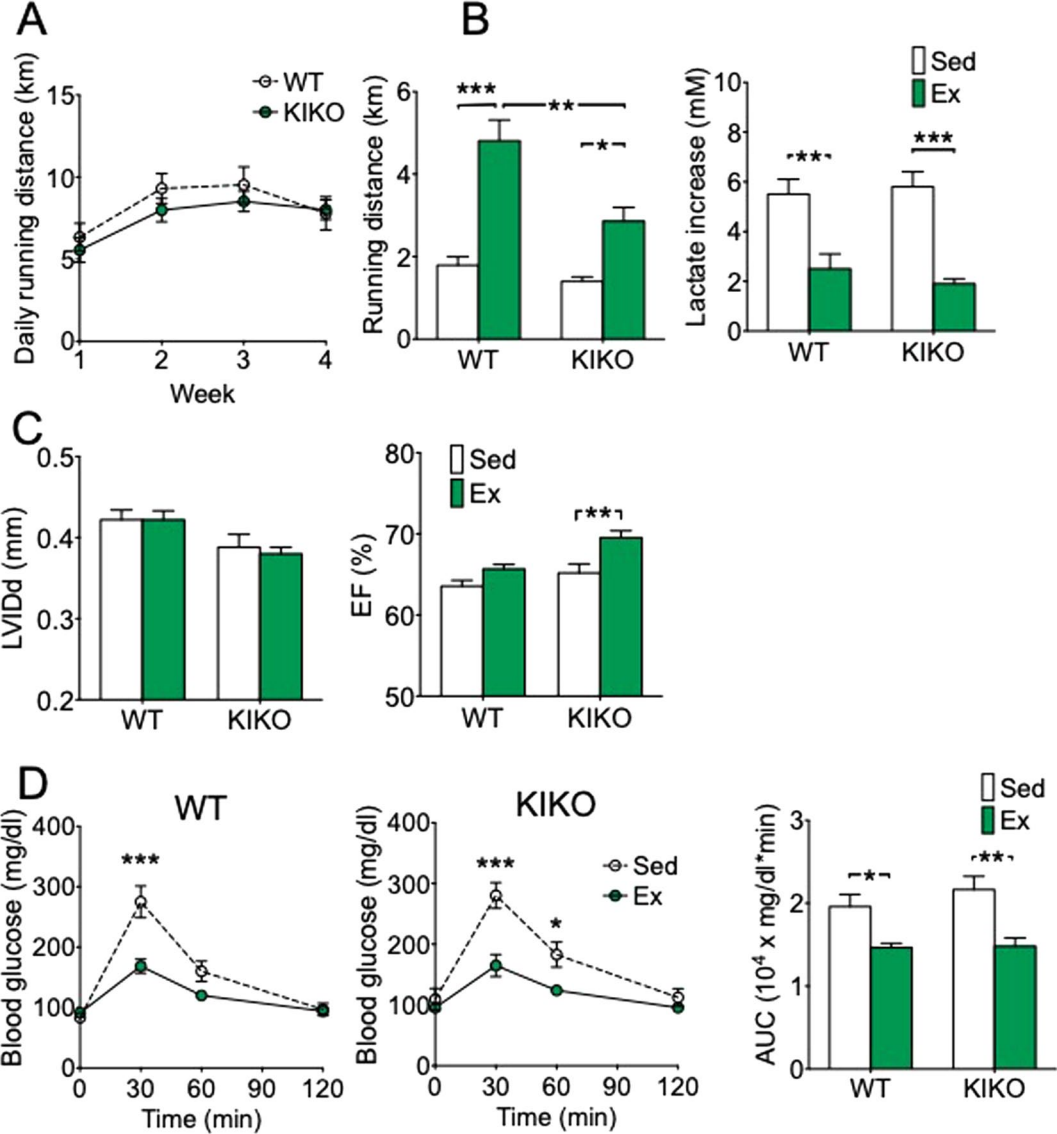
KIKO mice adapt to voluntary running normally. KIKO mice (4 month of age) were subjected to voluntary wheel running (Ex) (n = 8) or sedentary cage activity (Sed) (n = 8) for 4 weeks followed by treadmill running, Echo and GTT tests. (**A**) Voluntary running activity (daily running distance) in KIKO and WT mice; (**B**) Running distance and blood lactate increase by treadmill running test; (**C**) Echocardiography measurements of left ventricular internal diameter end diastole (LVIDd) and ejection fraction (EF); (**D**) Blood glucose levels during GTT and the area under the curve (AUC). *P < 0.05; **P < 0.01; ***P < 0.001.

An outstanding question whether KIKO mice have normal functional adaptations in response to endurance exercise training. To this end, we subjected KIKO mice to voluntary wheel running training (4 weeks), which has been shown to induce muscle function and metabolic adaptations^22–25^, starting at 2 or 4 months of age before they developed detectable functional deficits. Voluntary running activity (daily running distance) was not statistically different between KIKO and WT mice for both the age groups. Sedentary KIKO mice had normal running capacity compared with WT mice (Fig. 2B and Supplemental Fig. S2B), and 4 weeks of voluntary running improved exercise capacity in KIKO mice for both 2- and 4-month age groups (Fig. 2B and Supplemental Fig. S2B). Voluntary running for 4 weeks reduced blood lactate increase upon exhaustion in both WT and KIKO mice (Fig. 2B and Supplemental Fig. S2B). Interestingly, 4-month-old KIKO mice showed less significant improvement in running capacity than age-matched WT mice following exercise training (Fig. 2B), which may suggest reduced trainability in KIKO mice at this age.

Heart is one of the most affected organs/tissues in FRDA^7,8,26^, and regular exercise/physical activity improves cardiac function in healthy individuals^27^. It is not known whether endurance exercise training will improve cardiac function in FRDA. Here, we assessed the impact of long-term voluntary running on cardiac function in KIKO mice. We detected a general trend (not statistically significant) of decreased LVIDd in KIKO mice compared with WT mice for both ages and found no significant changes in this parameter following voluntary running training (Fig. 2C and Supplemental Fig. S2C). On the contrary, we detected significantly improved ejection fraction and fractional shortening in exercise trained KIKO mice compared with sedentary KIKO mice (Fig. 2C and Supplemental Fig. S2C). There was no sign of ECG abnormalities in KIKO mice with/without exercise training (Supplemental Table 3). These findings suggest that KIKO mice have normal cardiac adaptation to exercise training at 2 and 4 months of age.

**Table 3:** Impact of long-term exercise training on cardiac function in WT and KIKO mice.

	4 months		6 months	
	Sed	Ex	Sed	Ex
	(n = 4)	(n = 5)	(n = 4)	(n = 5)
IVSd (mm)	0.087 ± 0.010	0.091 ± 0.008	0.096 ± 0.009	0.091 ± 0.008
LVIDd (mm)	0.367 ± 0.011	0.390 ± 0.016	0.385 ± 0.021	0.395 ± 0.004
PWd (mm)	0.078 ± 0.007	0.061 ± 0.006	0.074 ± 0.013	0.069 ± 0.008
IVSs (mm)	0.129 ± 0.011	0.133 ± 0.006	0.138 ± 0.013	0.139 ± 0.008
LVIDs (mm)	0.255 ± 0.008	0.252 ± 0.013	0.261 ± 0.016	0.255 ± 0.003
PWs (mm)	0.119 ± 0.006	0.098 ± 0.009	0.109 ± 0.007	0.115 ± 0.008
PWT (%)	55.2 ± 10.9	62.6 ± 6.1	55.2 ± 17.9	69.4 ± 9.7
FS (%)	30.5 ± 0.3	35.5 ± 0.6***	32.2 ± 0.6	35.5 ± 0.5**
EF (%)	66.4 ± 0.5	73.2 ± 0.8***	68.8 ± 0.9	73.2 ± 0.6**

IVS, interventricular septal thickness; LVID, left ventricular internal dimension; PW, posterior wall thickness; d, end diastole; s, end systole; PWT, posterior wall thickening; EF, ejection fraction; FS, fractional shortening. Values are presented as mean ± SE. ** and *** denote *p* < 0.01 and *p* < 0.001 (vs. Sed group), respectively.

Voluntary running resulted in significantly reduced blood glucose at 30 min time point in the GTT and reduced AUC in both WT and *KIKO* mice compared with sedentary mice (*p* < 0.05) (Fig. 2D and Supplemental Fig. S2D). These findings show clear evidence of metabolic adaptation in response to endurance exercise training in KIKO mice with equal adaptability to that of WT mice.

### Long-term voluntary running prevents symptomatic onset of FRDA in KIKO mice

**Figure 3:**
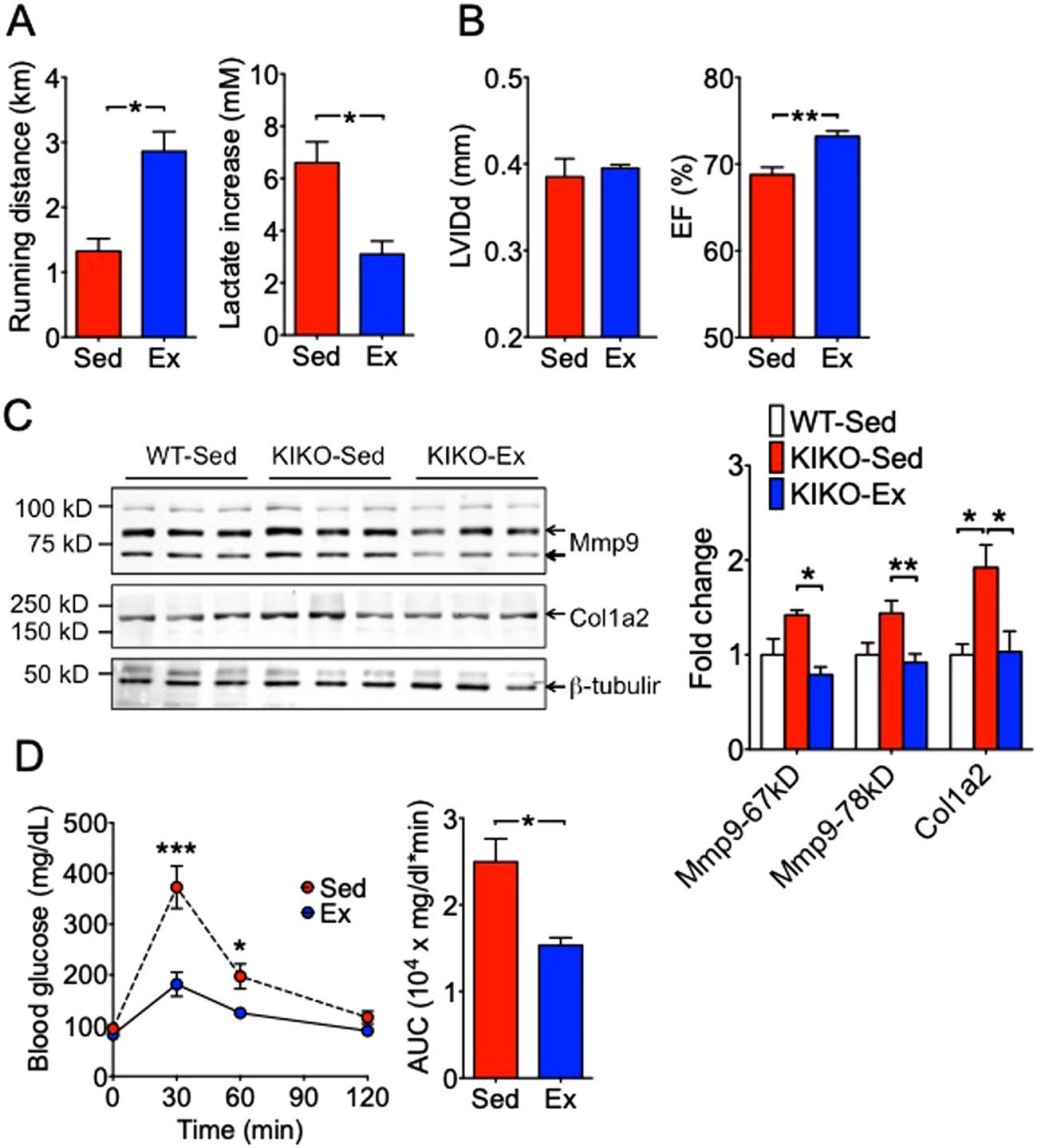
Long-term voluntary running completely prevents onset of exercise intolerance, cardiac dysfunction and glucose intolerance with significantly improved functions. KIKO mice (2 months of age) were subjected to voluntary wheel running (Ex) (n = 5) or sedentary cage activity (Sed) (n = 4) for 4 months followed by treadmill running, Echo and GTT tests. (**A**) Running distance and blood lactate increase by treadmill running test; (**B**) Echocardiography measurements of left ventricular internal diameter end diastole (LVIDd) and ejection fraction (EF); (**C**) Representative western blot images and quantification of Mmp9 and Col1a2 proteins in the left ventricle tissue comparing to wild type control (n = 4); (**D**) Blood glucose levels during GTT and the area under the curve (AUC). *P < 0.05; **P < 0.01; ***P < 0.001.

To determine if endurance exercise training prevents the onset of symptomatic FRDA in KIKO, we subjected KIKO mice to voluntary running for 4 months, starting at 2 months of age. This allowed investigation into the potential protective effects of long-term endurance exercise on KIKO mice at a young age that is equivalent to 7-years of age in humans before the onset of symptomatic FRDA. We observed that 6-month old exercise-trained KIKO mice achieved a running distance of 2.86 ± 0.30 km, which was more than 2-fold longer than the sedentary KIKO mice (1.32 ± 0.19 km; p < 0.05) (Fig. 3A). This running distance is also greater than that of healthy, sedentary WT mice (1.81 ± 0.07) (Fig. 1B). Meanwhile, the increase of blood lactate in exercise-trained KIKO mice was significantly reduced compared with sedentary KIKO mice (Fig. 3A). Similar findings were observed during the training period at 4 months of age (after 2 months of training) (Supplemental Fig. S3A). Taken together, these results show that long-term endurance exercise training starting at a young age in KIKO mice can completely prevent the onset of exercise intolerance and even improve exercise capacity.

To determine the impact of long-term endurance exercise on cardiac function in KIKO mice, we performed echocardiogram at 4 and 6 month of age. We did not observe a significant difference in LVIDd between sedentary and exercise-trained KIKO mice, but there was clear evidence of improved cardiac systolic function as shown by increased ejection fraction and fractional shortening after 2 and 4 months of training (Fig. 3B, Supplemental Fig. S3B and Table 3). To further confirm the impact of long-term endurance exercise in the heart in KIKO mice, we measured collagen I (Col1a2) and matrix metalloproteinase 9 (Mmp9) expression. Col1a2 is the most abundant protein of connective tissues, and Mmp9 plays a role in the deposition of extracellular matrix (ECM). Elevated expression of these proteins would be indicative of cardiac fibrosis. Col1a2 was moderately elevated in sedentary KIKO mice compared with age-matched WT control, which was completely normalized by long-term voluntary running (Fig. 3C). Similarly, Mmp9 proteins (both 67-kD and 78-kD bands) showed a trend of increased expression, which were completely normalized by exercise training (Fig. 3C). These findings collectively suggest that long-term exercise training prevents the deleterious cardiac maladaptation in KIKO mice.

We ascertained the metabolic benefit of exercise training in KIKO mice by GTT (Fig. 3C and Supplemental Fig. S3C). Exercise-trained KIKO mice showed significant lower blood glucose levels during GTT (at 30 and 60 min) and reduced AUC compared with sedentary KIKO mice (Fig. 3C). Similar findings were obtained after 2 months of training (Supplemental Fig. S3C). These findings, for the first time, show that long-term voluntary running in KIKO mice (starting at 2 months of age) completely prevents the onset of glucose intolerance.

### Long-term voluntary running promotes Irp1 expression and mitochondrial function without restoring Fxn expression

**Figure 4:**
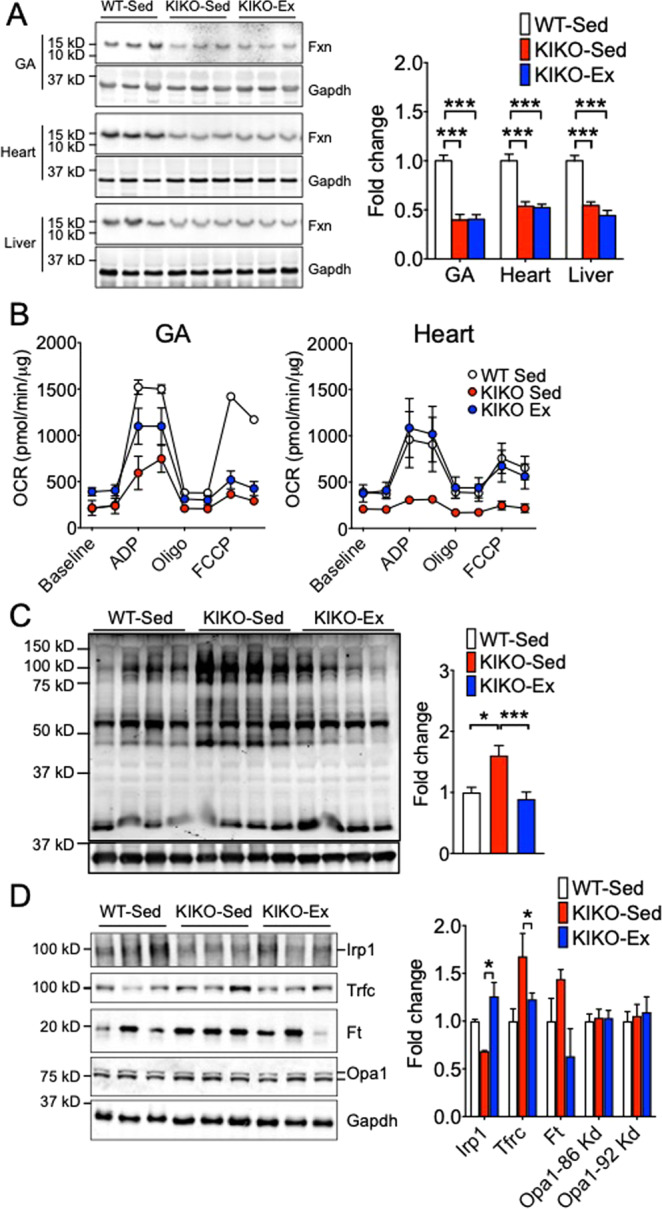
Long-term voluntary running promotes Irp1 expression and mitochondrial function in the absence of restored Fxn expression. KIKO mice (2 months of age) were subjected to voluntary wheel running (Ex) (n = 5) or sedentary cage activity (Sed) (n = 4) for 4 months. (**A**) Representative western blot images and quantification of Fxn protein in gastrocnemius muscle (GA), heart and liver using wild type mice (WT) (n = 4) as control; (**B**) Oxygen consumption rate (OCR) normalized to mitochondrial protein in isolated mitochondria from GA muscle and heart using Seahorse mitochondrial stress test. Baseline OCR was measured prior to addition of ADP, oligomycin (Oligo) and uncoupler, FCCP; (**B**) for measurement of maximal capacity and finally addition of antimycin A and rotenone (**C**) for non-mitochondrial OCR; (**C**) Representative western blot image and quantification of 4-HNE in gastrocnemius muscle; and (**D**) Representative western blot images and quantification of Irp1, Tfrc, Ft and Opa1 proteins in gastrocnemius muscle. *P < 0.05 and ***P < 0.001.

The pathologies of FRDA are caused by GAA expansion in the *FXN* gene^11,28^ that leads to primary and/or secondary defects, such as reduced frataxin expression, deficits in mitochondrial respiratory chain proteins containing ISCs, iron overload, and oxidative stress^14,15^. To gain mechanistic insight into the protective effects of endurance exercise training in KIKO mice, we measured frataxin, Cox4, electron transport chain complexes (CI-V), antioxidant enzymes in skeletal muscle, heart and liver by western blot. Our hypothesis was that long-term endurance exercise would restore Fxn expression in some or all these tissues. To our surprise, there was no sign of restoration of Fxn expression in any of these tissues (Fig. 4A), suggesting that long-term endurance exercise bypasses the need of restoring Fxn expression in preventing the pathologies of FRDA. We did not observe significant declines of any of the mitochondrial proteins and antioxidant enzymes in these tissues in KIKO mice except a trend of decreased Cox4 in the liver, nor did we observe significant impact of exercise (Supplemental Fig. S4A,B). On the contrary, when we assessed the respiratory function in isolated mitochondria from gastrocnemius muscle or heart, sedentary KIKO mice had significantly reduced state 3 respiration compared with WT mice, which was significantly prevented by long-term voluntary running (Fig. 4B). Furthermore, we observed significant increases of 4-Hydroxynonenal (4-HHE) in sedentary KIKO mouse skeletal muscle, which were completely mitigated by long-term voluntary running. These findings provide the first direct evidence of improved mitochondrial function along with reduced oxidative stress by long-term exercise training in a mouse model of FRDA.

Although the exact function of Fxn is not fully understood, Fxn appears to help assemble of iron-sulfur (Fe-S) cluster molecules that are critical for mitochondrial respiratory function^13^. In light of findings of improved mitochondrial function in the absence of restored Fxn expression, we explored the impact of endurance exercise training on other regulatory proteins involved in Fe-S biogenesis. In particular, we were interested in iron regulatory protein 1 (Irp1), a key regulator of cellular iron metabolism that has recently been shown to sustain mitochondrial iron supply and function in the absence of Fxn^29^. Consistent with this notion, we found that deficiency of Fxn led to a reduction of Irp1 expression in skeletal muscle and heart (Fig. 4C and Supplemental Fig. S4C), which were abolished by endurance exercise training. Sedentary KIKO mice had a significantly elevated and a trend of increased expression of transferrin receptor (Tfrc) and ferritin (Ft), respectively, which were prevented by endurance exercise training (Fig. 4C). A speculative notion is that endurance exercise promotes Irp1 expression, which may bypass Fxn in sustaining mitochondrial iron supply and function and abolishing the compensatory increases of Tfrc and Ft expression. Loss of FXN expression in FRDA leads to the generation of oxidative stress, mitochondrial energy imbalance and an increase in lipid peroxidation, which have been shown in cerebellar granule neurons and mouse fibroblasts^14,30^. Western blot of 4-HNE showed increased signal in skeletal muscle sedentary KIKO mice, which was absent in exercise-trained KIKO mice. These findings confirmed that exercise training prevents oxidative stress in FRDA.

## Discussion

The major findings of this study include: (1) KIKO mice had an age-dependent development of exercise intolerance, cardiac dysfunction and metabolic abnormality; (2) Young KIKO mice had normal functional adaptations in response to 4 weeks of endurance exercise training, including improved exercise capacity, glucose metabolism and cardiac function; (3) Long-term voluntary running starting early in life prevented symptomatic onset of FRDA in KIKO mice, raising the possibility of using endurance exercise training to prevent the onset of FRDA in early diagnosed patients; and (4) Long-term voluntary running improved mitochondrial function in skeletal muscle and heart in the absence of restored Fxn expression, but with evidence of restored expression of Irp1 as well as reduced oxidative stress in skeletal muscle.

Clinical symptoms in FRDA patients appear during childhood or adolescence, and are characterized by a progressive and relentless ataxia, hypertrophic cardiomyopathy, with approximately one third of those affected developing diabetes or carbohydrate intolerance^4,12,31^. Patients typically require wheelchairs within 10 to 20 years after symptoms begin, and their life expectancy is shortened to an average of 40–50 years^32^, mostly because of congestive heart failure and arrhythmias^31^. In this study, KIKO mice were found to have reduced Fxn expression early in life, but did not develop exercise intolerance, glucose intolerance and moderate cardiac dysfunction until 6 months of age, thus demonstrating an age-dependent development of organ dysfunction, which is consistent with the disease progression in FRDA patients.

It is well established that endurance exercise training promotes cardiac function, glucose metabolism, and exercise capacity^22,27^. In this regard, a prescribed exercise program in FRDA may prove to be beneficial, and a study in an appropriate animal model of FRDA with physiological exercise is warranted. Up to date, there have not been a study evaluating the effectiveness of endurance exercise training on FRDA in animal models or in human patients. Here, we demonstrate that voluntary wheel running in KIKO mice improves locomotive, cardiac and metabolic functions and that the underlying mechanism is not related to restoration of Fxn expression, but normalized iron protein expression and reduced oxidative stress possibly through enhanced Irp1 expression. To our knowledge, this is the first study showing a profound protective effect in mitigating age-related physiological dysfunction in a genetic model of FRDA in mice.

Current strategies for the treatment of FRDA mainly focused on correcting frataxin expression, which intervenes the pathogenetic cascade downstream of frataxin deficiency, enhance antioxidant expression, and even some further gene or protein replacement or cellular therapies^6,33^. In fact, no therapies have been proven to cure or slow disease progression effectively^34^. Here, we found that young KIKO mice have the same adaptability as WT mice. Furthermore, long-term voluntary running starting at a young age completely prevented symptomatic onset in KIKO mice at 6 months of age. These findings may imply that FRDA patients could benefit from endurance exercise training if started early. These findings of the benefits of long-term endurance exercise is extremely exciting and raise the possibility of mitigating the onset of the disease or at least slowing disease progression with exercise intervention in FRDA patients. This is particularly true if we implement genetic screening is implemented so to identify affected individuals before the onset of the symptoms. However, at present, we do not know if exercise with certain intensity and duration can reverse the disease process.

Mechanistically, our data revealed that the benefits of exercise were not due to restored Fxn expression, but that deteriorated mitochondrial function and increased oxidative stress could be prevented/corrected. It has been shown that enhanced Irp1 expression can normalized iron sulfate cluster function in the absence of Fxn and protects mitochondrial function^29^. Future research should address the question if enhanced Irp1 expression or activity could prevent the onset of and/or correct the functional abnormalities in KIKO mice, which might be of tremendously clinical values to FRDA patients.

In summary, we have shown that a mouse genetic model of FRDA (KIKO mice) develops exercise intolerance, glucose intolerance as well as moderate cardiac dysfunction in an age-dependent manner at about 6 months of age. These abnormalities do not appear caused by loss of mitochondria and reduced antioxidant defense, but impaired mitochondrial respiratory function along with increased oxidative stress. Long-term endurance exercise starting at a young age completely prevented the functional abnormalities in KIKO mice along with enhanced Irp1 expression and improved mitochondrial function and reduced oxidative stress in the absence of restored Fxn expression. These findings make endurance exercise training an appealing therapeutic for FRDA patients, particularly at the early phase of the disease.

## Material and Methods

### Animals

All animal procedures were conducted in accordance with the protocols that had been approved by the University of Virginia Institutional Animal Care and Use Committee. Frataxin knock-in/knock-out (KIKO, Jackson Laboratory, Stock#014162, B6.Cg) mice harbor one allele of the frataxin (GAA)_230_ expansion mutation (*Fxn*^*tm1Pand*^) on one chromosome (KI), and one allele of the frataxin exon 4-deleted mutation (*Fxn*^*tm1Mkn*^) on the other chromosome (KO). We crossbred *Fxn*^*tm1Mkn*^*Fxn*^*tm1Pand*^ with homozygous *Fxn*^*tm1Pand*^ mice (Jackson Laboratory, Stock#008470) to obtain *Fxn*^*tm1Mkn*^*Fxn*^*tm1Pand*^ mice (KIKO) for the studies. To obtain wild type control mice (WT) with close genetic background, we bred *Fxn*^*tm1Pand*^ mice with C57BL/6 mice for two rounds to obtain second generation C57BL/6 wild type mice (WT; one generation apart from the KIKO mice) as control. All mice were housed in temperature-controlled (22 °C) quarters with a 12:12-h light-dark cycle with free access to water and normal chow (Harlan 7912). Hindlimb muscles, including soleus (SO), plantaris (PL), gastrocnemius (GA), heart and liver were harvested after the mice were humanely sacrificed under anesthesia and weighed and flash frozen in liquid nitrogen for storage at −80 °C before further analyses.

### Genotyping

Mouse genomic DNA was isolated from tail, and PCR-based genotyping for KIKO was performed according to the instruction provided by Jackson Laboratory.

### Voluntary wheel running

Mice were individually housed in cages equipped with a running wheel in a designated room with a dark-light cycle (6:00 pm off, 6:00 am on) as previously described^35^.

### Glucose tolerance tests (GTTs)

GTT was performed on 6 hours-fasted mice by measuring glucose using a glucose meter (Ascensia, Bayer) for the tail vein blood following intraperitoneal injection of glucose (2.0 g/kg body weight)^25^.

### Electrocardiography (ECG)

ECG was performed on fully conscious mice using the ECGenie (Mouse Specifics, Boston, MA) as described previously^36^.

### Echocardiography (Echo)

Echo was performed with measurements of LV end-diastolic and end-systolic diameters, end-diastolic and end-systolic wall thicknesses of interventricular septum, end-diastolic and end-systolic LV posterior wall thickness. Fractional shortening and ejection fraction were calculated as described^36^.

### Treadmill running test

Treadmill running test protocol with blood lactate measures before and after running was performed as described previously^37^.

### *In vivo* muscle function

Maximal isometric torque of the plantar flexor muscles was assessed using the Aurora Scientific 305 C Dual-Mode Muscle Lever System as previously described^38^. To account for differences in body size among mice during longitudinal studies torque was normalized by body mass (g).

### Western blot

The detailed procedure has been described^24^. The following primary antibodies were used: anti-Fxn (GeneTex, Cat.#: GTX54036), anti-Cox4 (Cell Signaling Technology, Cat.#: 48445), total OXPHOS Rodent WB Antibody Cocktail (MitoSciences, Cat.#: MS604), anti-Sod1 (Abcam, Cat.#: ab16831), anti-Sod2 (Enzo Life Sciences, Cat.#: ADI-SOD-111-D), anti-Sod3 (R&D Systems, Cat.#: AF4817), anti-Cat (Abcam, Cat.#: ab15834), anti-β-Tublin (Cell Signaling Technology, Cat.#: 2146 S), anti-Mmp9 (Proteintech, Cat.#: 10375-2-AP), anti-Col1a2 (Proteintech, Cat.#: 14695-1-AP), anti-Irp1 (Santa Cruz Biotechnology, Cat.#: sc-166022), anti-Tfrc (Abcam, Cat.#: ab84036), anti-Ft (Abcam, Cat.#: ab75973), anti-Opa1 (Novus Biological, Cat.#: NBP2-34206), anti-4-HNE (Abcam, Cat.#: ab48506) and anti-Gapdh (Cell Signaling Technology, Cat.#: 14C10). The proteins were detected and quantified using an Odyssey Infrared Imaging System (LI-COR Biosciences, Lincoln, NE).

### Mitochondrial isolation and oxygen consumption

Mitochondria-enriched fractions from gastrocnemius muscle and heart were isolated via differential centrifugation in the presence of protease (Roche Diagnostics, Cat# 11836153001) and phosphatase inhibitors (Sigma, #P5726, P0044) as previously described^22^. Mitochondrial respiration was measured using a seahorse XF-24 Flux Analyzer (Seahorse Biosciences, Billerica, MA) as previously described^39^. Briefly, pellets of isolated mitochondrial fraction from sedentary and exercise-trained KIKO mice and sedentary WT mice were resuspended in Mitochondria Assay Solution (MAS; 70 mM sucrose, 220 mM mannitol, 10 mM KH_2_PO_3_, 5 mM MgCl_2_, 2 mM HEPES, 1 mM EGTA, and 0.2% (w/v) fatty-acid free BSA (pH 7.2) at 37 °C. Protein concentration was determined using BCA assay (ThermoFisher Scientific, Waltham, MA), and 2.5 µg of protein were adhered to Seahorse XF24 Cell Culture Microplate by 2,000 × g centrifugation for 20 minutes at 4 °C. Then, 450 µL of 1X MAS with 10 mM pyruvate and 2 mM malate added to each well, and the plate was warmed to 37 °C in a CO_2_-free incubator for 20 minutes. Coupled respiration was measured in sequential additions of the following substrates; 5 mM ADP, 5 µM oligomycin, and 7 µM FCCP to evaluate State 3, State 4, leak and maximal respiration, respectively.

### Statistical analysis

All data were presented as mean ± SEM. For comparisons between two experimental groups, two-tailed Student’s *t*-test was performed. For comparisons among multiple experimental groups, one-way ANOVA followed by the Student Newman-Kuels post hoc test was performed. Two-way ANOVA was used to analyze the two main effects (genotype and Ex) and possible interaction. Tukey post hoc tests were performed if significant (*P* < 0.05) main effects or interactions were found. Values of *P* < 0.05 were considered statistically significant.

## Supplementary information

Supplemental tables, figures and figure legends.
