# Author Correction: Long-term voluntary running prevents the onset of symptomatic *Friedreich’s ataxia* in mice

**DOI:** 10.1038/s41598-021-96112-1

**Published:** 2021-08-09

**Authors:** Henan Zhao, Bevan M. Lewellen, Rebecca J. Wilson, Di Cui, Joshua C. Drake, Mei Zhang, Zhen Yan

**Affiliations:** 1 Department of Medicine, University of Virginia School of Medicine, Charlottesville, VA 22908 USA; 2 Department of Pharmacology, University of Virginia School of Medicine, Charlottesville, VA 22908 USA; 3 Department of Molecular Physiology and Biological Physics, University of Virginia School of Medicine, Charlottesville, VA 22908 USA; 4 Center for Skeletal Muscle Research at Robert M. Berne Cardiovascular Research Center, University of Virginia School of Medicine, Charlottesville, VA 22908 USA; 5 Dalian Medical University, Dalian, 116044 Liaoning China

Correction to: *Scientific Reports* https://doi.org/10.1038/s41598-020-62952-6, published online 08 April 2020

The original version of this Article contained an error in the spelling of the author Bevan M. Lewellen which was incorrectly given as Beven M. Lewellen. The original Article and accompanying Supplementary Information file have been corrected.

