# Supplementary material for: Long-term voluntary running prevents the onset of symptomatic Friedreich’s ataxia in mice
Source: Sci Rep. 2020 Apr 8;10:6095. doi: 10.1038/s41598-020-62952-6 (PMC7142077; doi:10.1038/s41598-020-62952-6)

Long-term voluntary running prevents the onset of symptomatic *Friedreich's ataxia* in mice

Henan Zhao,[4,5] Bevan M. Lewellen,[4] Rebecca J. Wilson,[4] Di Cui,[4] Joshua C. Drake,[4] Mei Zhang,[1,4] and Zhen Yan[1,2,3,4,*]

Departments of Medicine[1], Pharmacology[2], Molecular Physiology and Biological Physics[3], and Center for Skeletal Muscle Research at Robert M. Berne Cardiovascular Research Center[4], Charlottesville, Virginia 22908, USA; Dalian Medical University[5], Dalian, Liaoning 116044, China

*Correspondence: Zhen Yan, Ph.D., 409 Lane Rd., MR4–6031A, Charlottesville, VA 22908, USA. E-mail: zhen.yan@virginia.edu

Supplemental Figure Legends

Supplemental Fig. S1. General characterization of KIKO mice. Fxn protein in different tissues, body weight, tibia length and muscle contractile function were measured in KIKO and WT mice at 2 (n =14-21), 4 (n = 13-25) and 6 months of age (n = 18-36). A) PCR-based genotyping of tail DNA from KIKO and WT mice; B) Representative western blot images and quantification of Fxn protein in GA, heart and liver; C) Body weight and tibia length measurements; D) twitch contraction and tetanic contraction of plantar flexor muscles normalized by body weight in vivo; E) D) Blood glucose levels during GTT and the area under the curve (AUC); F) Representative western blot images and quantification of Cox4, complex I (CI), complex II (CII), complex III (CIII), and complex V (CV) in GA, heart and liver. $^{**}P <$ 0.01 and $^{***}P < 0.001$.

Supplemental Fig. S2. KIKO mice adapt to 4 weeks of voluntary running. KIKO mice (2 month of age) were subjected to voluntary wheel running (Ex) (n = 4) or sedentary cage activity (Sed) (n = 3) for 4 weeks followed by treadmill running, Echo and GTT tests. A) Voluntary running activity (daily running distance) in KIKO and WT mice; B) Running distance and blood lactate increase by treadmill running test; C) Echocardiography measurements of left ventricular internal diameter end diastole (LVIDd) and ejection fraction (EF); D) Blood glucose levels during GTT and the area under the curve (AUC). $^{*}P < 0.05$ and $^{***}P < 0.001$.

Supplemental Fig. S3. Improved exercise capacity, cardiac function and metabolism after

long-term voluntary running. KIKO mice (2 months of age) were subjected to voluntary wheel running (Ex) (n = 5) or sedentary cage activity (Sed) (n = 4) for 2 months followed by treadmill running, Echo and GTT tests. A) Running distance and blood lactate increase by treadmill running test; B) Echocardiography measurements of left ventricular internal diameter end diastole (LVIDd) and ejection fraction (EF); C**)** Blood glucose levels during GTT and the area under the curve (AUC). $^{*}P < 0.05$ and $^{***}P < 0.001$.

Supplemental Fig. S4. Mitochondrial respiratory protein and antioxidant enzyme expression in skeletal muscle, heart and liver in KIKO mice after long-term voluntary running. KIKO mice (2 months of age) were subjected to voluntary wheel running (Ex) (n = 5) or sedentary cage activity (Sed) (n = 4) for 2 months followed by western blotting analysis. A) Representative western blot images and quantification of Cox4, mitochondrial complex I (CI), complex II (CII), complex III (CIII), and complex V (CV) in GA, heart and liver; B) Representative western blot images and quantification of Sod1, Sod2, Sod3 and Cat in GA, heart and liver; and D) Representative western blot images and quantification of Irp1, Tfrc, Ft and Opa1 proteins in the heart. $^{*}P < 0.05$

Table S1. KIKO mice have normal muscle and heart weight at 6 months of age

Genotype	Tibia length (mm)	SO (mg/mm)	PL (mg/mm)	GA (mg/mm)	Heart (mg/mm)
WT (n = 36)	18.3 ± 0.1	0.46 ± 0.01	0.88 ± 0.03	6.42 ± 0.15	6.04 ± 0.19
KIKO (n = 18)	18.1 ± 0.1*	0.47 ± 0.02	0.90 ± 0.05	6.29 ± 0.27	5.81 ± 0.28

SO, soleus; PL; plantaris; GA, gastrocnemius. Values are presented at mean ± SE. * denotes $p < 0.05$ (vs. WT).

Supplemental Table S2. KIKO mice have normal electrocardiogram

	2 months		4 months		6 months	
	WT (n = 21)	KIKO (n =14)	WT (n = 13)	KIKO (n = 25)	WT (n = 36)	KIKO (n =15)
HR (bpm)	702.3 ± 13.2	679.5 ± 12.8	705.3 ± 16.5	686.4 ± 9.9	734.9 ± 5.8	713.6 ± 10.5
HRVar	37.0 ± 4.7	34.8 ± 5.7	25.8 ± 3.6	27.4 ± 3.1	18.5 ± 2.0	28.7 ± 6.3
PR (ms)	28.3 ± 0.7	28.0 ± 0.6	29.4 ± 0.7	28.2 ± 1.2	27.6 ± 0.6	26.8 ± 0.9
QRS (ms)	12.3 ± 0.3	12.6 ± 0.5	11.9 ± 0.3	12.2 ± 0.2	12.7 ± 0.3	11.6 ± 0.4
QT (ms)	44.4 ± 0.8	45.3 ± 0.9	45.6 ± 1.1	45.4 ± 0.4	44.2 ± 0.4	43.3 ± 1.0

HR, heart rate (bpm, beats/min); HRVar, heart rate variability; PR, PR interval; QRS, QRS complex; QT, QT interval. Values are presented as mean ± SE.

Supplemental Table 3. Electrocardiogram in WT and KIKO mice at 2 or 4 months of age following 4-week voluntary running

	WT		KIKO	
	Sed	Ex	Sed	Ex
	2 months of age			
	(n = 13)	(n = 8)	(n = 7)	(n = 7)
HR (bpm)	714.9 ± 12.0	702.0 ± 13.0	692.0 ± 22.7	686.4 ± 9.9
HRVar	23.4 ± 5.0	35.5 ± 5.1	38.7 ± 8.1	33.4 ± 6.0
PR (ms)	28.2 ± 0.9	28.5 ± 1.1	28.8 ± 0.8	28.8 ± 0.8
QRS (ms)	12.0 ± 0.3	12.8 ± 0.6	13.1 ± 0.4	11.7 ± 0.3
QT (ms)	43.4 ± 0.5	45.4 ± 0.8	46.2 ± 0.7	45.9 ± 1.2
	4 months of age			
	(n = 6)	(n = 6)	(n = 6)	(n = 7)
HR (bpm)	713.8 ± 20.8	722.8 ± 17.7	677.0 ± 42.1	704.4 ± 15.6
HRVar	26.9 ± 13.8	29.6 ± 6.1	40.1 ± 10.8	34.5 ± 5.2
PR (ms)	26.7 ± 1.9	29.6 ± 0.9	28.5 ± 1.5	28.1 ± 1.7
QRS (ms)	11.4 ± 0.7	12.1 ± 0.8	11.8 ± 0.8	12.3 ± 0.5
QT (ms)	44.0 ± 1.2	44.8 ± 0.8	45.1 ± 2.2	45.5 ± 0.8

HR, heart rate (bpm, beats/min); HRVar, heart rate variability; PR, PR interval; QRS, QRS complex; QT, QT interval. Values are presented as mean ± SE.

Supplemental Fig. S1

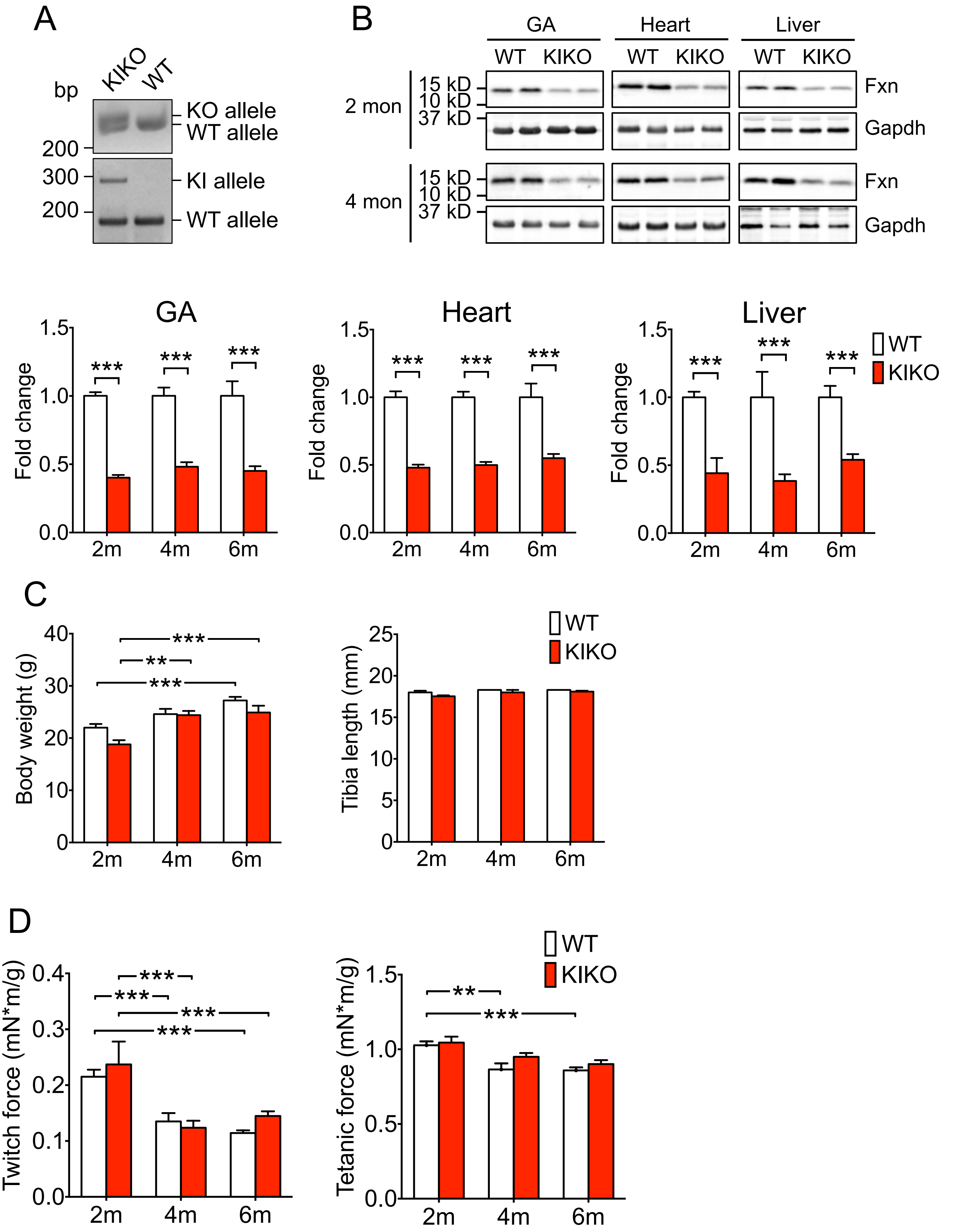

Supplemental Fig. S1 (continued)

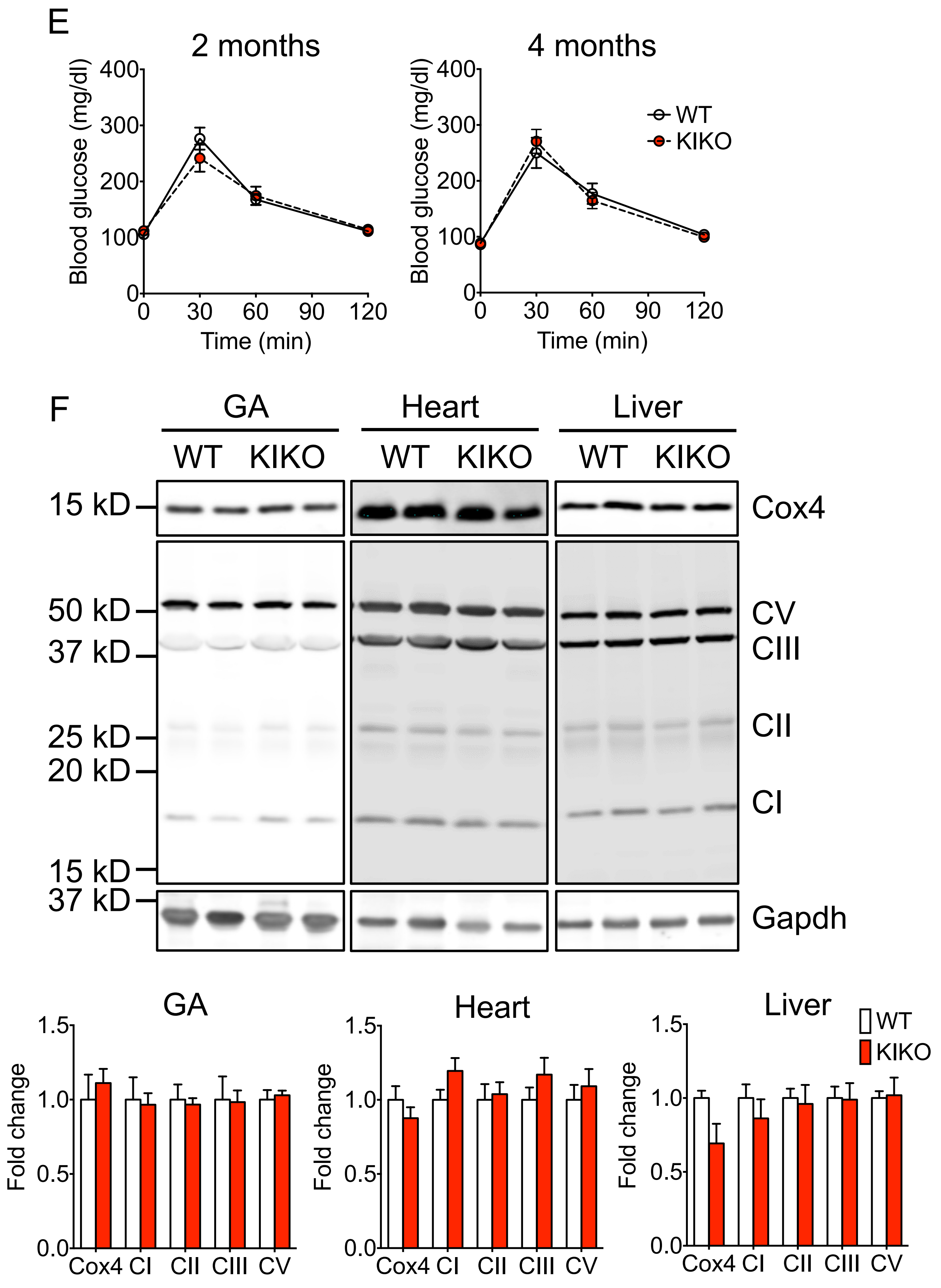

Supplemental Fig. S2

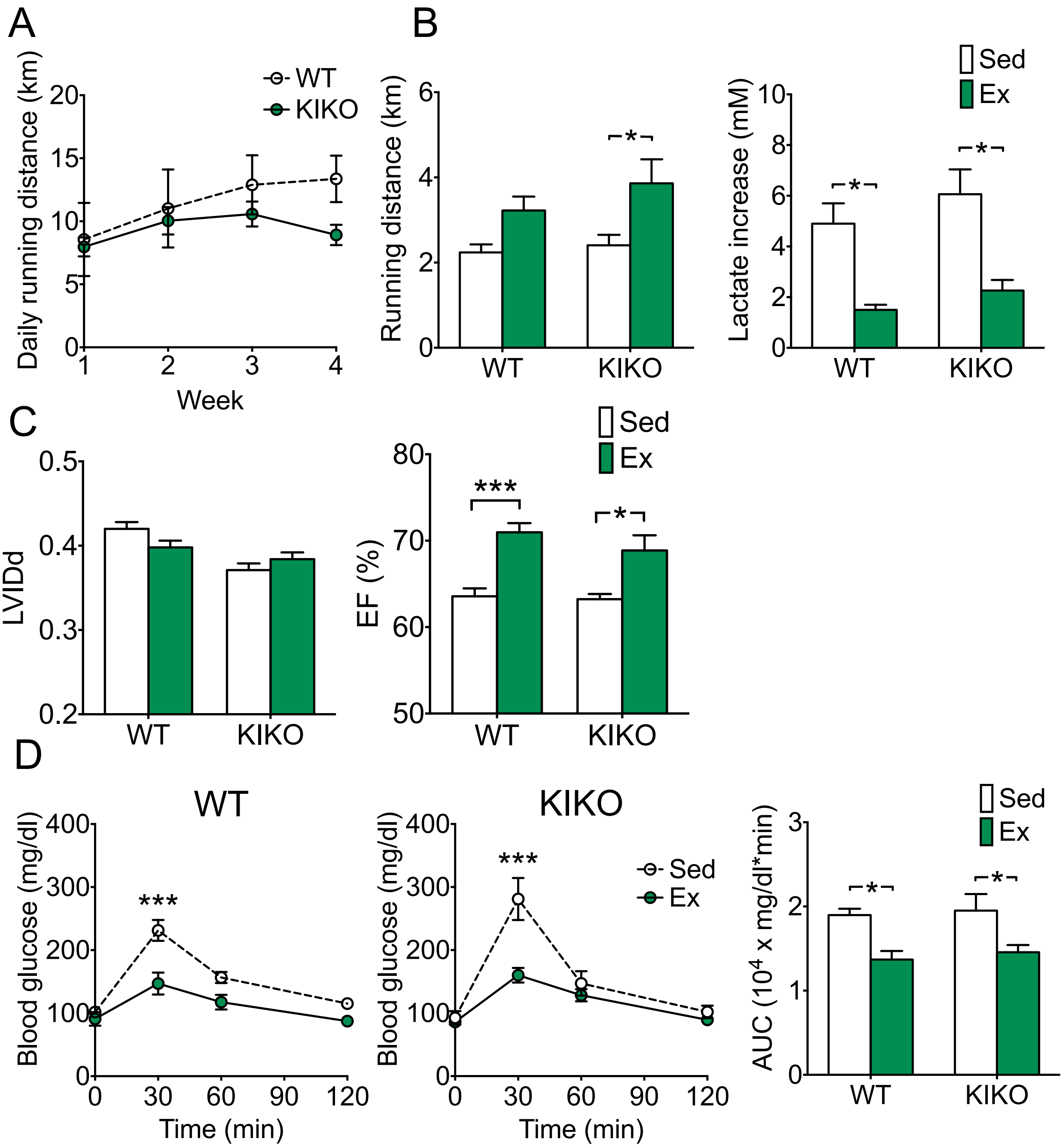

Supplemental Fig. S3

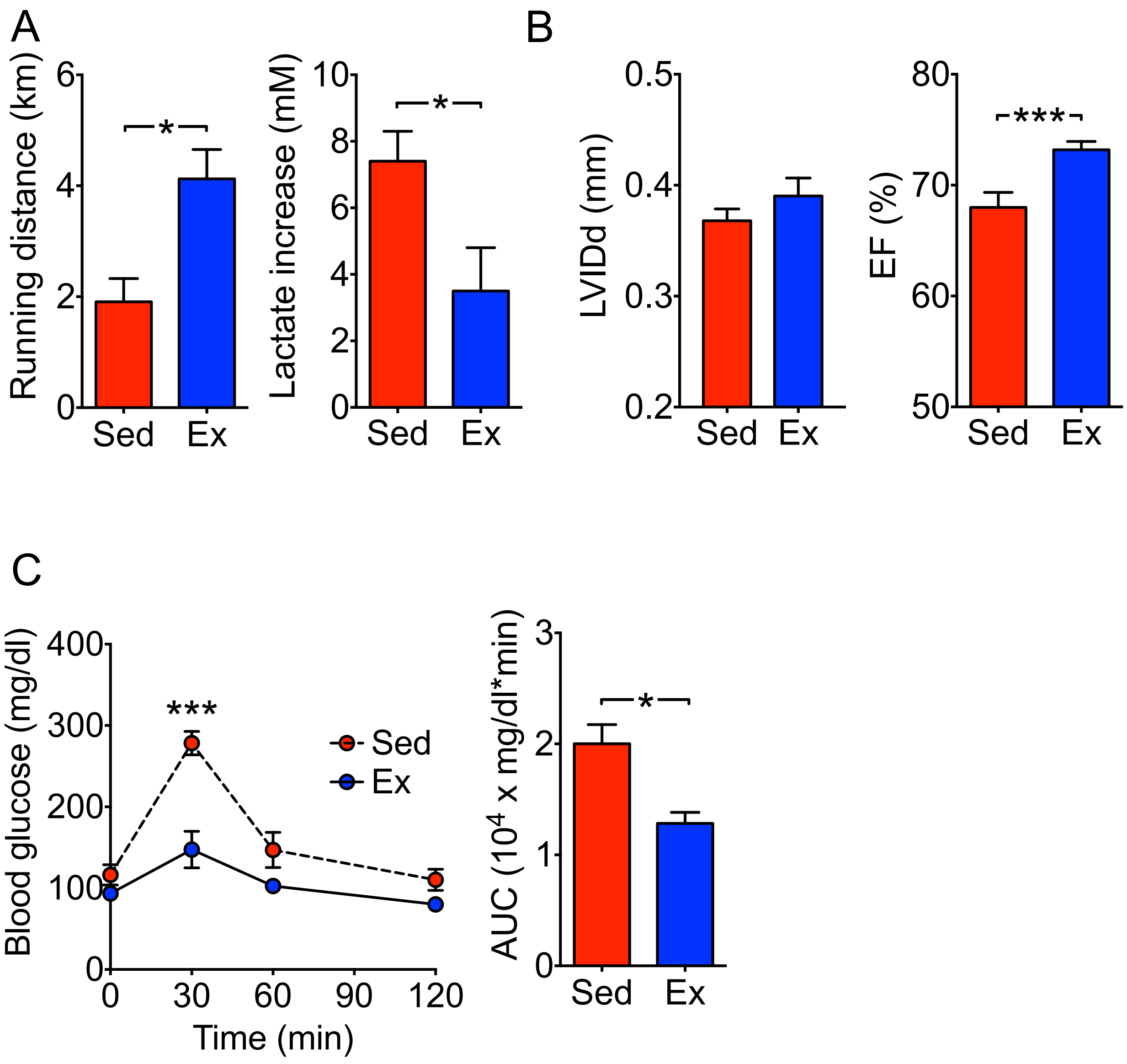

Supplemental Fig. S4

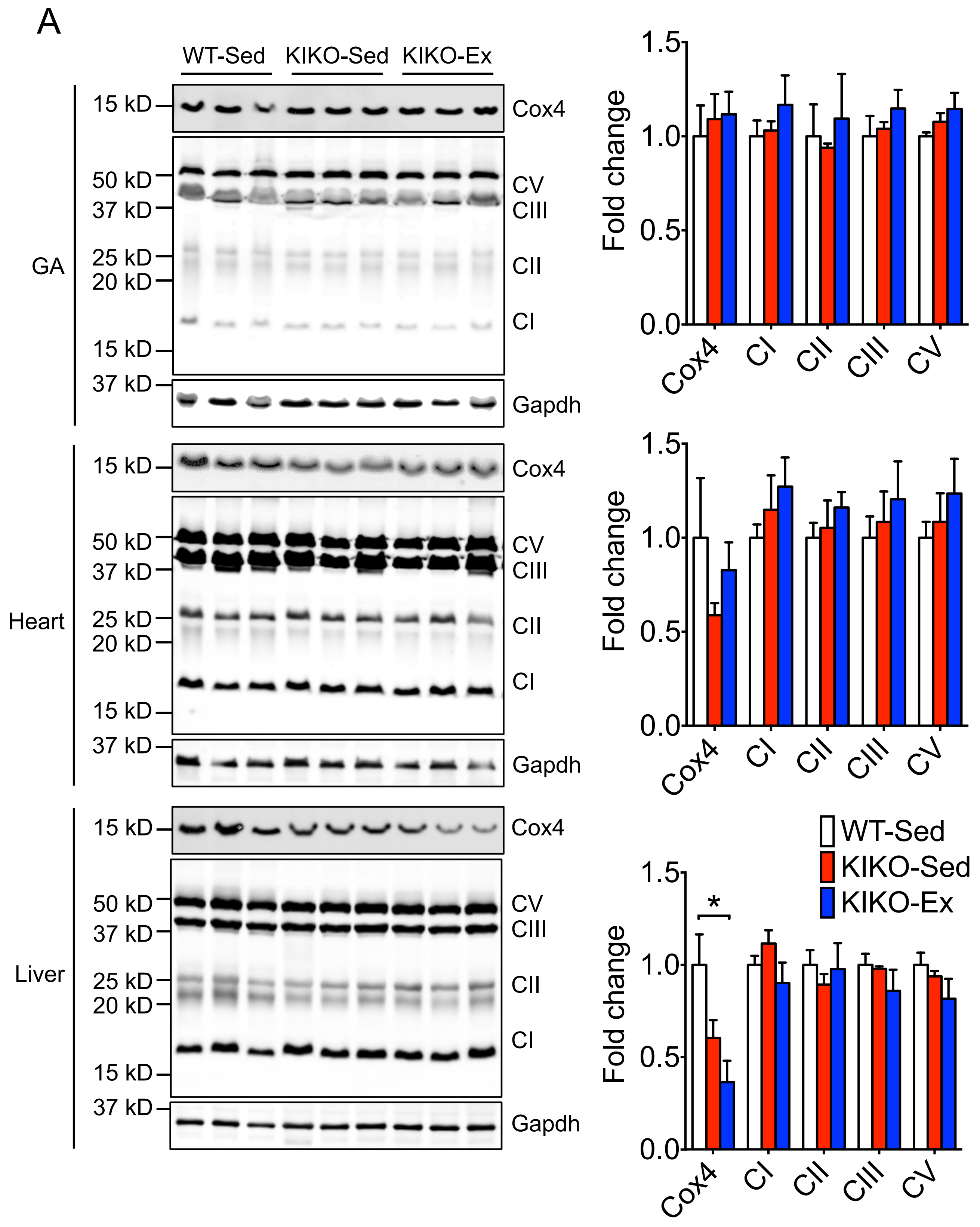

Supplemental Fig. S4 (continued)

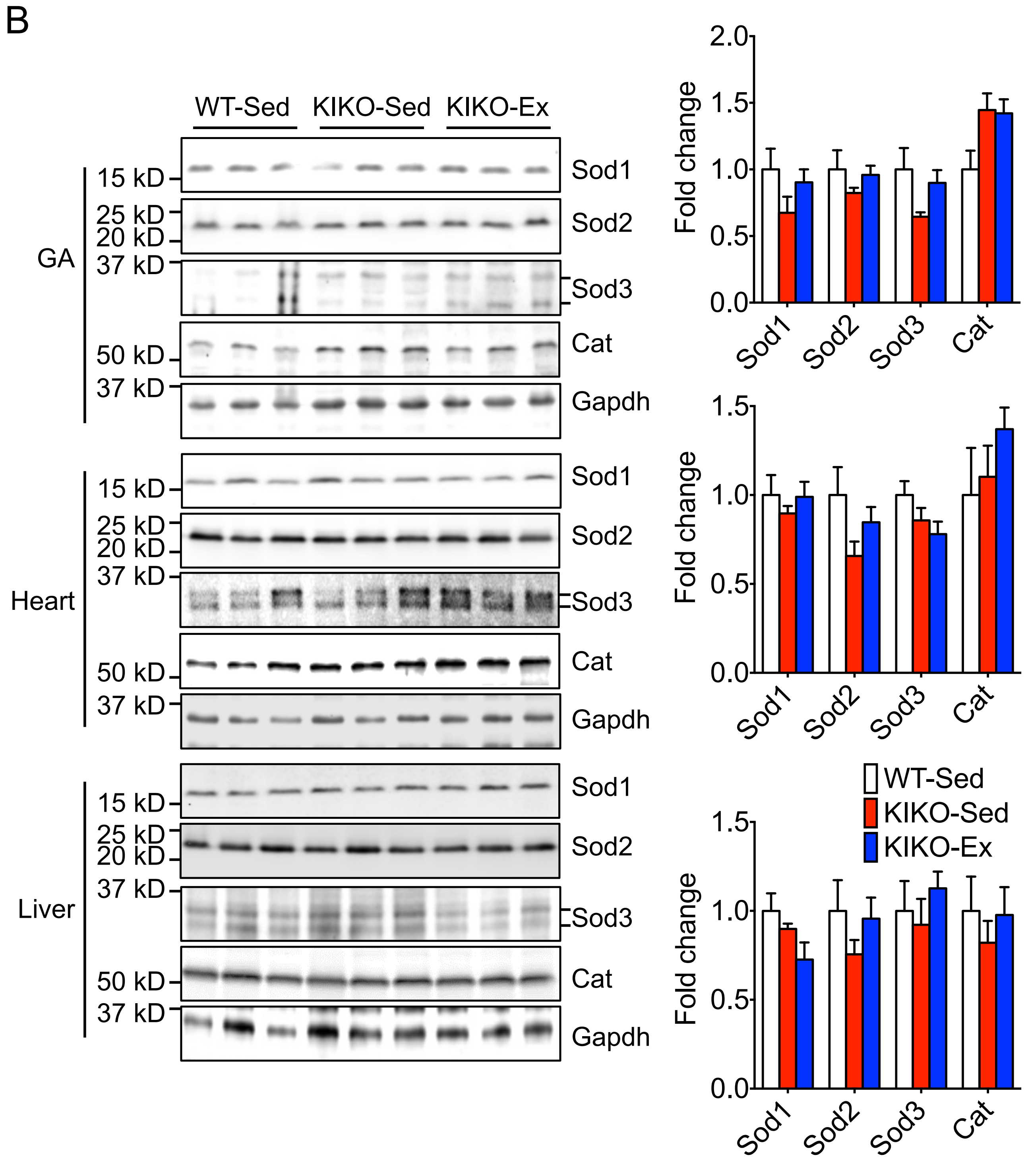

Supplemental Fig. S4 (continued)

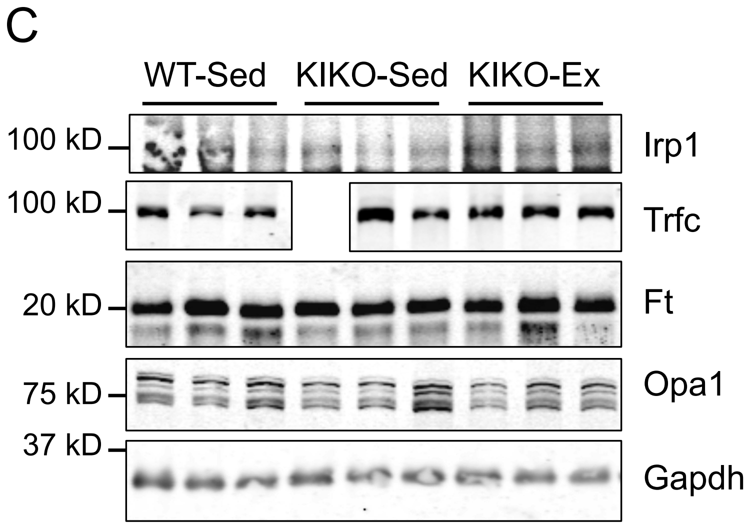

Supplement: Supplementary file 1 — Supplemental tables, figures and figure legends. [file 41598_2020_62952_MOESM1_ESM.pdf]
